# Supplementary material for: The resource elasticity of control
Source: eLife. 2026 Nov 6;14:RP105507. doi: 10.7554/eLife.105507 (PMC13743078; doi:10.7554/eLife.105507)
Supplement: Supplementary file 1 [file elife-105507-mdarchecklist1.docx]

**Materials Design Analysis Reporting (MDAR)**

**Checklist for Authors**

The [MDAR framework](https://osf.io/xfpn4/) establishes a minimum set of requirements in transparent reporting mainly applicable to studies in the life sciences.

*eLife* asks authors to **provide detailed information within their article** to facilitate the interpretation and replication of their work. Authors can also upload supporting materials to comply with relevant reporting guidelines for health-related research (see [EQUATOR Network](http://www.equator-network.org/%20)), life science research (see the [BioSharing Information Resource](http://biosharing.org/)), or animal research (see the [ARRIVE Guidelines](http://www.plosbiology.org/article/info:doi/10.1371/journal.pbio.1000412) and the [STRANGE Framework](https://doi.org/10.1038/d41586-020-01751-5); for details, see *eLife*’s [Journal Policies](https://reviewer.elifesciences.org/author-guide/journal-policies)). Where applicable, authors should refer to any relevant reporting standards materials in this form.

For all that apply, please note **where in the article** the information is provided. Please note that we also collect information about data availability and ethics in the submission form.

**Materials:**

| **Newly created materials** | **Indicate where provided: section/figure legend** | **N/A** |
| --- | --- | --- |
| The manuscript includes a dedicated "materials availability statement" providing transparent disclosure about availability of newly created materials including details on how materials can be accessed and describing any restrictions on access. |  | X |
|  |  |  |
| **Antibodies** | **Indicate where provided: section/figure legend** | **N/A** |
| For commercial reagents, provide supplier name, catalogue number and [RRID](https://scicrunch.org/resources), if available. |  | X |
|  |  |  |
| **DNA and RNA sequences** | **Indicate where provided: section/figure legend** | **N/A** |
| Short novel DNA or RNA including primers, probes: Sequences should be included or deposited in a public repository. |  | X |
|  |  |  |
| **Cell materials** | **Indicate where provided: section/figure legend** | **N/A** |
| Cell lines: Provide species information, strain. Provide accession number in repository OR supplier name, catalog number, clone number, OR RRID. |  | X |
| Primary cultures: Provide species, strain, sex of origin, genetic modification status. |  | X |
|  |  |  |
| **Experimental animals** | **Indicate where provided: section/figure legend** | **N/A** |
| Laboratory animals or Model organisms: Provide species, strain, sex, age, genetic modification status. Provide accession number in repository OR supplier name, catalog number, clone number, OR RRID. |  | X |
| Animal observed in or captured from the field: Provide species, sex, and age where possible. |  | X |
|  |  |  |
| **Plants and microbes** | **Indicate where provided: section/figure legend** | **N/A** |
| Plants: provide species and strain, ecotype and cultivar where relevant, unique accession number if available, and source (including location for collected wild specimens). |  | X |
| Microbes: provide species and strain, unique accession number if available, and source. |  | X |
|  |  |  |
| **Human research participants** | **Indicate where provided: section/figure legend) or state if these demographics were not collected** | **N/A** |
| If collected and within the bounds of privacy constraints report on age, sex, gender and ethnicity for all study participants. | Methods, "Participants". Age (range 18-45, mean 33, SE 0.6) and sex (38% female) are reported. Gender and ethnicity were not collected. |  |

**Design:**

| **Study protocol** | **Indicate where provided: section/figure legend** | **N/A** |
| --- | --- | --- |
| If the study protocol has been pre-registered, provide DOI. For clinical trials, provide the trial registration number OR cite DOI. | Methods, "Pre-registration". Both studies were pre-registered prior to data collection: <https://aspredicted.org/Q7M_BHR> (Initial study) and <https://aspredicted.org/CHW_12H> (Replication study). No trial registration number applies (not a clinical trial). |  |
|  |  |  |
| **Laboratory protocol** | **Indicate where provided: section/figure legend** | **N/A** |
| Provide DOI OR other citation details if detailed step-by-step protocols are available. |  | X |
|  |  |  |
| **Experimental study design (statistics details) *** | | |
| **For in vivo studies: State whether and how the following have been done** | **Indicate where provided: section/figure legend. If it could have been done, but was not, write “not done”** | **N/A** |
| Sample size determination | Methods, "Power analysis". Done. Initial study (N = 264): Bayesian power analysis (Kruschke, 2014), aiming for a credible-interval width under 0.1 for the opt-in probability, based on pilot data (N = 19). A bootstrap of the pilot data gave over 98% power for the main regression effects. Replication study (N = 250): bootstrap resampling of the Initial dataset, aiming for 95% power to detect a significant canonical correlation. |  |
| Randomisation | Methods, "Planet types" and "Experimental Task". Done. Each participant was randomly assigned one planet from each of the three conditions (low, high inelastic and high elastic controllability), and the order of planets was randomized. There were no between-participant groups. |  |
| Blinding | Not done. Single-group design with no experimenter-delivered conditions. All conditions were run automatically online. |  |
| Inclusion/exclusion criteria | Methods, "Participants" and "Self-report measures". Done. Prolific screening: high engagement in prior studies, English fluency, no psychiatric diagnoses, English-speaking western countries, ages 18-45. Pre-registered exclusions: more than 8 comprehension-quiz mistakes, under 90% vehicle-choice accuracy, and opting out in 90% or more of trials (22 and 23 excluded), plus infrequency-item removals (11). |  |
|  |  |  |
| **Sample definition and in-laboratory replication** | **Indicate where provided: section/figure legend** | **N/A** |
| State number of times the experiment was replicated in the laboratory. | Results (first paragraph) and Methods, "Power analysis". The experiment was run in two independent studies: an Initial study (N = 264) and a pre-registered Replication study (N = 250). Within each study, participants completed 30 trips on each of four planets. |  |
| Define whether data describe technical or biological replicates. | Methods, "Regression analyses" and "Individual differences". Data are biological replicates: each data point comes from an independent human participant. Repeated trials within a participant are not treated as independent replicates. They are modeled with participant-level random effects (mixed-effects regressions). |  |
|  |  |  |
| **Ethics** | **Indicate where provided: section/submission form** | **N/A** |
| Studies involving human participants: State details of authority granting ethics approval (IRB or equivalent committee(s), provide reference number for approval. | Methods, "Participants" and submission form. The study was approved the Hebrew University Ethics Committee (approval number 2022-06213). Participants gave written informed consent. |  |
| Studies involving experimental animals: State details of authority granting ethics approval (IRB or equivalent committee(s), provide reference number for approval. |  | X |
| Studies involving specimen and field samples: State if relevant permits obtained, provide details of authority approving study; if none were required, explain why. |  | X |
|  |  |  |
| **Dual Use Research of Concern (DURC)** | **Indicate where provided: section/submission form** | **N/A** |
| If study is subject to dual use research of concern regulations, state the authority granting approval and reference number for the regulatory approval. |  | X |

**Analysis:**

| **Attrition** | **Indicate where provided: section/figure legend** | **N/A** |
| --- | --- | --- |
| Describe whether exclusion criteria were pre-established. Report if sample or data points were omitted from analysis. If yes, report if this was due to attrition or intentional exclusion and provide justification. | The exclusion criteria were pre-established and pre-registered before data collection. Participants were intentionally excluded (not lost to attrition) for: (1) inattentiveness, i.e. more than 8 mistakes on the comprehension quizzes, (2) less than 90% accuracy in choosing the vehicle matching the treasure's destination, indicating poor task comprehension, and (3) opting out in 90% or more of trials, indicating lack of engagement. This excluded 22 participants (8.3%) from the Initial study and 23 (8.9%) from the Replication study. In addition, 11 participants (Initial: 6, Replication: 5) were removed for failing infrequency items, following best-practice recommendations for detecting inattentive responding. The first 15 trips on each planet were treated as a learning phase and excluded from the choice analyses, following the pre-registered plan. |  |
|  |  |  |
| **Statistics** | **Indicate where provided: section/figure legend** | **N/A** |
| Describe statistical tests used and justify choice of tests. | Methods, "Regression analyses", "Individual differences", "Model comparison", "Canonical Correlation Analysis (CCA)"; Figure 3, 5 and 6 legends. Opting in (binary) and extra tickets purchased (ordinal) were analyzed with mixed-effects logistic and cumulative probit regressions, which suit these outcomes and account for repeated trips within participants (Wald Z tests; Shapiro-Wilk for normality). Computational models were compared with the integrated BIC (log Bayes factors), which penalizes model complexity. Associations between model parameters and psychopathology used robust canonical correlation analysis (Spearman), because psychopathology measures share substantial variance. Significance was assessed with permutation tests (1,000 iterations), which make no distributional assumptions. Sample sizes were set by Bayesian and bootstrap power analyses ("Power analysis"). |  |
|  |  |  |
| **Data availability** | **Indicate where provided: section/submission form** | **N/A** |
| For newly created and reused datasets, the manuscript includes a data availability statement that provides details for access (or notes restrictions on access). | Data availability statement and submission form. The behavioral data from both studies (Initial, N = 264; Replication, N = 250) are available at <https://github.com/lsolomyak/human_inference_of_elastic_control/tree/master/data>. No previously published datasets were reused. |  |
| When newly created datasets are publicly available, provide accession number in repository OR DOI and licensing details where available. | The data are available in the GitHub repository (see above) under the Creative Commons Attribution 4.0 International license (CC BY 4.0), see the LICENSE file in the data folder. No restrictions on access. |  |
| If reused data is publicly available provide accession number in repository OR DOI, OR URL, OR citation. | No previously published datasets were reused. |  |
|  |  |  |
| **Code availability** | **Indicate where provided: section/figure legend** | **N/A** |
| For any computer code/software/mathematical algorithms essential for replicating the main findings of the study, whether newly generated or re-used, the manuscript includes a data availability statement that provides details for access or notes restrictions. | Data availability statement in the manuscript. It states that the task code, analysis code and computational models are publicly available at <https://github.com/lsolomyak/human_inference_of_elastic_control>, with no restrictions on access. |  |
| Where newly generated code is publicly available, provide accession number in repository, OR DOI OR URL and licensing details where available. State any restrictions on code availability or accessibility. | The task, the computational models (controllability and elastic controllability models, fitting and simulation routines) and the analysis scripts are available at <https://github.com/lsolomyak/human_inference_of_elastic_control> under the MIT License. No restrictions on availability or access. |  |
| If reused code is publicly available provide accession number in repository OR DOI OR URL, OR citation. | Analyses used the publicly available R packages "ordinal" (mixed-effects logistic and probit regressions), "lme4" (individual-differences regressions) and "ccAPP" (robust canonical correlation analysis), cited in Methods. |  |

**Reporting:**

The MDAR framework recommends adoption of discipline-specific guidelines, established and endorsed through community initiatives.

| **Adherence to community standards** | **Indicate where provided: section/figure legend** | **N/A** |
| --- | --- | --- |
| State if relevant guidelines (e.g., ICMJE, MIBBI, ARRIVE, STRANGE) have been followed, and whether a checklist (e.g., CONSORT, PRISMA, ARRIVE) is provided with the manuscript. |  | X |

* We provide the following guidance regarding transparent reporting and statistics; we also refer authors to [Ten common statistical mistakes to watch out for when writing or reviewing a manuscript](https://doi.org/10.7554/eLife.48175).

**Sample-size estimation**

- You should state whether an appropriate sample size was computed when the study was being designed
- You should state the statistical method of sample size computation and any required assumptions
- If no explicit power analysis was used, you should describe how you decided what sample (replicate) size (number) to use

**Replicates**

- You should report how often each experiment was performed
- You should include a definition of biological versus technical replication
- The data obtained should be provided and sufficient information should be provided to indicate the number of independent biological and/or technical replicates
- If you encountered any outliers, you should describe how these were handled
- Criteria for exclusion/inclusion of data should be clearly stated
- High-throughput sequence data should be uploaded before submission, with a private link for reviewers provided (these are available from both GEO and ArrayExpress)

**Statistical reporting**

- Statistical analysis methods should be described and justified
- Raw data should be presented in figures whenever informative to do so (typically when N per group is less than 10)
- For each experiment, you should identify the statistical tests used, exact values of N, definitions of center, methods of multiple test correction, and dispersion and precision measures (e.g., mean, median, SD, SEM, confidence intervals; and, for the major substantive results, a measure of effect size (e.g., Pearson's r, Cohen's d)
- Report exact p-values wherever possible alongside the summary statistics and 95% confidence intervals. These should be reported for all key questions and not only when the p-value is less than 0.05.

**Group allocation**

- Indicate how samples were allocated into experimental groups (in the case of clinical studies, please specify allocation to treatment method); if randomization was used, please also state if restricted randomization was applied
- Indicate if masking was used during group allocation, data collection and/or data analysis
