# Supplementary material for: The resource elasticity of control
Source: eLife. 2026 Nov 6;14:RP105507. doi: 10.7554/eLife.105507 (PMC13743078; doi:10.7554/eLife.105507)
Supplement: Peer review history

# Editor report: eLife Assessment

**DOI:** 10.7554/eLife.105507.4.sa0

**Authors:** Roshan Cools

**Affiliations:** Donders Institute for Brain, Cognition and Behaviour, Radboud University Nijmegen Netherlands

**Keywords:** Incomplete, Solid, Valuable

This study makes the **valuable** claim that people track, specifically, the elasticity of control (that is, the degree to which outcome depends on how many resources - such as money - are invested), and that control elasticity is impaired in certain types of psychopathologies. A novel task is introduced that provides **solid** evidence that this learning process occurs and that human behavior is sensitive to changes in the elasticity of control. Evidence that elasticity inference is distinct from more general learning mechanisms and is related to psychopathology remains **incomplete**.

# Referee report: Reviewer #1 (Public review)

**DOI:** 10.7554/eLife.105507.4.sa1

Summary:

The authors investigated the elasticity of controllability by developing a task that manipulates the probability of achieving a goal with a baseline investment (which they refer to as inelastic controllability) and the probability that additional investment would increase the probability of achieving a goal (which they refer to as elastic controllability). They found that a computational model representing the controllability and elasticity of the environment accounted better for the data than a model representing only the controllability. They also found that prior biases about the controllability and elasticity of the environment was associated with a composite psychopathology score. The authors conclude that elasticity inference and bias guide resource allocation.

Strengths:

This research takes a novel theoretical and methodological approach to understanding how people estimate the level of control they have over their environment, and how they adjust their actions accordingly. The task is innovative and both it and the findings are well-described (with excellent visuals). They also offer thorough validation for the particular model they develop. The research has the potential to theoretically inform understanding of control across domains, which is a topic of great importance.

Weaknesses:

In its revised form, the manuscript addresses most of my previous concerns. The main remaining weakness pertains to the analyses aimed at addressing my suggesting of Bayesian updating as an alternative to the model proposed by the authors. My suggestion was to assume that people perform a form of function approximation to relate resource expenditure to success probability. The authors performed a version of this where people were weighing evidence for a few canonical functions (flat, step, linear), and found that this model underperforms theirs. However, this Bayesian model is quite constrained in its ability to estimate the function relating resources. A more robust test would be to assume a more flexible form of updating that is able to capture a wide range of distributions (e.g., using basis functions, gaussian processes, or nonparametric estimators); see, e.g., work by Griffiths on human function learning. The benefit of testing this type of model is that it would make contact with a known form of inference that individuals engage in across various settings, and therefore could offer a more parsimonious and generalizable account of function learning, whereby learning of resource elasticity is a special case. I defer to the authors as to whether they'd like to pursue this direction, but if not I think it's still important that they acknowledge that they are unable to rule out a more general process like this as an alternative to their model. This also pertains to inferences about individual differences, which currently hinge on their preferred model being the most parsimonious.

# Referee report: Reviewer #2 (Public review)

**DOI:** 10.7554/eLife.105507.4.sa2

Summary:

In this paper, the authors test whether controllability beliefs and associated actions/resource allocation are modulated by things like time, effort, and monetary costs (what they call "elastic" as opposed to "inelastic" controllability). Using a novel behavioral task and computational modeling, they find that participants do indeed modulate their resources depending on whether they are in an "elastic," "inelastic," or "low controllability" environment. The authors also find evidence that psychopathology is related to specific biases in controllability.

Strengths:

This research investigates how people might value different factors that contribute to controllability in a creative and thorough way. The authors use computational modeling to try to dissociate "elasticity" from "overall controllability," and find some differential associations with psychopathology. This was a convincing justification for using modeling above and beyond behavioral output, and yielded interesting results. Notably, the authors conclude that these findings suggest that biased elasticity could distort agency beliefs via maladaptive resource allocation. Overall, this paper reveals important findings about how people consider components of controllability.

Weaknesses:

The authors have gone to great lengths to revise the manuscript to clarify their definitions of "elastic" and "inelastic" and bolster evidence for their computational model, resulting in an overall strong manuscript that is valuable for elucidating controllability dynamics and preferences. One minor weakness is that the justification for the analysis technique for the relationships between the model parameters and the psychopathology measures remains lacking given the fact that simple correlational analyses did not reveal any significant associations nor were there results of any regression analyses. That said, the authors did preregister the CCA analysis, so while perhaps not the best method, it was justified to complete it. Regardless of method, the psychopathology results are not particularly convincing, but provide an interesting jumping-off point for further exploration in future work.

# Referee report: Reviewer #3 (Public review)

**DOI:** 10.7554/eLife.105507.4.sa3

A bias in how people infer the amount of control they have over their environment is widely believed to be a key component of several mental illnesses including depression, anxiety, and addiction. Accordingly, this bias has been a major focus in computational models of those disorders. However, all of these models treat control as a unidimensional property, roughly, how strongly outcomes depend on action. This paper proposes---correctly, I think---that the intuitive notion of "control" captures multiple dimensions in the relationship between action and outcome. In particular, the authors identify one key dimension: the degree to which outcome depends on how much *effort* we exert, calling this dimension the "elasticity of control". They additionally argue that this dimension (rather than the more holistic notion of controllability) may be specifically impaired in certain types of psychopathology. This idea has the potential to change how we think about several major mental disorders in a substantial way, and can additionally help us better understand how healthy people navigate challenging decision-making problems. More concisely, it is a *very good idea*.

The more concrete contributions, however, are not as strong. In particular, evidence for the paper's most striking claims is weak. Quoting the abstract, these claims are (1) "the elasticity of control [is] a distinct cognitive construct guiding adaptive behavior" and (2) "overestimation of elasticity is associated with elevated psychopathology involving an impaired sense of control."

Main issues

I'll highlight the key points.

- The task cannot distinguish elasticity inference from general learning processes

- Participants were explicitly instructed about elasticity, with labeled examples

- The psychopathology claims rely on an invalid interpretation of CCA, and are contradicted by simple correlations (elasticity bias and the sense of agency scale is r=0.03)

Distinct construct

Starting with claim 1, there are three subclaims here. (1A) People's behavior is sensitive to differences in elasticity; (1B) there are mental processes specific to elasticity inference, i.e., not falling out of general learning mechanisms; and, implicitly, (1C) people infer elasticity naturally as they go about their daily lives. The results clearly support 1A. However, 1B and 1C are not well supported.

(1B) The data cannot support the "distinct cognitive construct" claim because the task is too simple to dissociate elasticity inference from more general learning processes (also raised by Reviewer 1). The key behavioral signature for elasticity inference (vs. generic controllability inference) is the transfer across ticket numbers, illustrated in Fig 4. However, this pattern is also predicted by a standard Bayesian learner equipped with an intuitive causal model of the task. Each ticket gives you another chance to board and the agent infers the probability that each attempt succeeds. Crucially, this logic is not at all specific to elasticity or even control. An identical model could be applied to inferring the bias of a coin from observations of whether any of N tosses were heads-a task that is formally identical to this one (at least, the intuitive model of the task; see first minor comment).

Importantly, this point cannot be addressed by showing that the author's model fits data better than this or any other specific Bayesian model. It is not a question of whether one particular updating rule explains data better than another. Rather, it is a question of whether the task can distinguish between biases in *elasticity* inference versus biases in probabilistic inference more generally. The present task cannot make this distinction because it does not make separate measurements of the two types of inference. To provide compelling evidence that elasticity inference is a "distinct cognitive construct", one would need to show that there are reliable individual differences in elasticity inference that generalize across contexts but do not generalize to computationally similar types of probabilistic inference (e.g. the coin flipping example).

(1C) The implicit claim that people infer elasticity outside of the experimental task is undermined by the experimental design. The authors explicitly tell people about the two notions of control as part of the training phase: "To reinforce participants' understanding of how elasticity and controllability were manifested in each planet, [participants] were informed of the planet type they had visited after every 15 trips."

In the revisions, the authors seem to go back and forth on whether they are claiming that people infer elasticity without instruction (I won't quote it here). I'll just note that the examples they provide in the most recent rebuttal are all cases in which one never receives explicit labels about elasticity. If people only infer elasticity when it is explicitly labeled, I struggle to see its relevance for understanding human cognition and behavior.

Psychopathology

Finally, I turn to claim 2, that "overestimation of elasticity is associated with elevated psychopathology involving an impaired sense of control." The CCA analysis is in principle unable to support this claim. As the authors correctly note in their latest rebuttal, the CCA does show that "there is a relationship between psychopathology traits and task parameters". The lesion analysis further shows that "elasticity bias specifically contributes to this relationship" (and similarly for the Sense of Agency scale). Crucially, however, this does *not* imply that there is a relationship between those two variables. The most direct test of that relationship is the simple correlation, which the authors report only in a supplemental figure: there is no relationship (r=0.03). Although it is of course possible that there is a relationship that is obscured by confounding variables, the paper provides no evidence-statistical or otherwise-that such a relationship exists.

Minor comments

The statistical structure of the task is inconsistent with the framing. In the framing, participants can make either one or two second boarding attempts (jumps) by purchasing extra tickets. The additional attempt(s) will thus succeed with probability p for one ticket and 2p - p^2 for two tickets; the p^2 captures the fact that you only take the second attempt if you fail on the first. A consequence of this is buying more tickets has diminishing returns. In contrast, in the task, participants always jumped twice after purchasing two tickets, and the probability of success with two tickets was exactly double that with one ticket. Thus, if participants are applying an intuitive causal model to the task, the researcher could infer "biases" in elasticity inference that are probably better characterized as effective use of prior information (encoded in the causal model).

The model is heuristically defined and does not reflect Bayesian updating. For example, it over-estimates maximum control by not using losses with less than 3 tickets (intuitively, the inference here depends on what your beliefs about elasticity). Including forced three-ticket trials at the beginning of each round makes this less of an issue; but if you want to remove those trials, you might need to adjust the model. The need to introduce the modified model with kappa is likely another symptom of the heuristic nature of the model updating equations.

# Author comment: Author response

**DOI:** 10.7554/eLife.105507.4.sa4

**Authors:** Levi Solomyak, Aviv Emanuel, Eran Eldar

**Affiliations:** Hebrew University of Jerusalem Jerusalem Israel; Hebrew University of Jerusalem Jerusalem Israel; The Hebrew University of Jerusalem Jerusalem Israel

The following is the authors’ response to the previous reviews

**Reviewer #1 (Public review):**

This research takes a novel theoretical and methodological approach to understanding how people estimate the level of control they have over their environment and how they adjust their actions accordingly. The task is innovative and both it and the findings are well-described (with excellent visuals). They also offer thorough validation for the particular model they develop. The research has the potential to theoretically inform understanding of control across domains, which is a topic of great importance.

We thank the Reviewer for their favorable appraisal and valuable suggestions, which have helped clarify and strengthen the study’s conclusion.

In its revised form, the manuscript addresses most of my previous concerns. The main remaining weakness pertains to the analyses aimed at addressing my suggesting of Bayesian updating as an alternative to the model proposed by the authors. My suggestion was to assume that people perform a form of function approximation to relate resource expenditure to success probability. The authors performed a version of this where people were weighing evidence for a few canonical functions (flat, step, linear), and found that this model underperformed theirs. However, this Bayesian model is quite constrained in its ability to estimate the function relating resources. A more robust test would be to assume a more flexible form of updating that is able to capture a wide range of distributions (e.g., using basis functions, gaussian processes, or nonparametric estimators); see, e.g., work by Griffiths on human function learning. The benefit of testing this type of model is that it would make contact with a known form of inference that individuals engage in across various settings and therefore could offer a more parsimonious and generalizable account of function learning, whereby learning of resource elasticity is a special case. I defer to the authors as to whether they'd like to pursue this direction, but if not I think it's still important that they acknowledge that they are unable to rule out a more general process like this as an alternative to their model. This pertains also to inferences about individual differences, which currently hinge on their preferred model being the most parsimonious.

We thank the Reviewer for this thoughtful suggestion. We acknowledge that more flexible function learning approaches could provide a stronger test in favor of a more general account. Our Bayesian model implemented a basis function approach where the weights of three archetypal functions (flat, step, linear) are learned from experience Testing models with more flexible basis functions would likely require a task with more than three levels of resource investment (1, 2, or 3 tickets). This would make an interesting direction for future work expanding on our current findings. We now incorporate this suggestion in more detail in our updated manuscript (335-341):

“Second, future models could enable generalization to levels of resource investment not previously experienced. For example, controllability and its elasticity could be jointly estimated via function approximation that considers control as a function of invested resources. Although our implementation of this model did not fit participants’ choices well (see Methods), other modeling assumptions drawn from human function learning [30] or experimental designs with continuous action spaces may offer a better test of this idea.”

**Reviewer #2 (Public review):**

This research investigates how people might value different factors that contribute to controllability in a creative and thorough way. The authors use computational modeling to try to dissociate "elasticity" from "overall controllability," and find some differential associations with psychopathology. This was a convincing justification for using modeling above and beyond behavioral output and yielded interesting results. Notably, the authors conclude that these findings suggest that biased elasticity could distort agency beliefs via maladaptive resource allocation. Overall, this paper reveals important findings about how people consider components of controllability. The authors have gone to great lengths to revise the manuscript to clarify their definitions of "elastic" and "inelastic" and bolster evidence for their computational model, resulting in an overall strong manuscript that is valuable for elucidating controllability dynamics and preferences.

We thank the Reviewer for their constructive feedback throughout the review process, which has substantially strengthened our manuscript and clarified our theoretical framework.

One minor weakness is that the justification for the analysis technique for the relationships between the model parameters and the psychopathology measures remains lacking given the fact that simple correlational analyses did not reveal any significant associations.

We note that the existence of bivariate relationships is not a prerequisite for the existence of multivariate relationships. Conditioning the latter on the former, therefore, would risk missing out on important relationships existing in the data. Ultimately, correlations between pairs of variables do not offer a sensitive test for the general hypothesis that there is a relationship between two sets of variables. As an illustration, consider that elasticity bias correlated in our data (r = .17, p<.001) with the difference between SOA (sense of agency) and SDS (self-rating depression). Notably, SOA and SDS were positively correlated (r = .47, p<.001), and neither of them was correlated with elasticity bias (SOA: r=.04 p=.43, SDS: r=-.06, p=.16). It was a dimension that ran between them that mapped onto elasticity bias. This specific finding is incidental and uncorrected for multiple comparisons, hence we do not report it in the manuscript, but it illustrates the kinds of relationships that cannot be accounted for by looking at bivariate relationships alone.

**Reviewer #3 (Public review):**

A bias in how people infer the amount of control they have over their environment is widely believed to be a key component of several mental illnesses including depression, anxiety, and addiction. Accordingly, this bias has been a major focus in computational models of those disorders. However, all of these models treat control as a unidimensional property, roughly, how strongly outcomes depend on action. This paper proposes---correctly, I think---that the intuitive notion of "control" captures multiple dimensions in the relationship between action and outcome.

In particular, the authors identify one key dimension: the degree to which outcome depends on how much *effort* we exert, calling this dimension the "elasticity of control". They additionally argue that this dimension (rather than the more holistic notion of controllability) may be specifically impaired in certain types of psychopathology. This idea has the potential to change how we think about several major mental disorders in a substantial way and can additionally help us better understand how healthy people navigate challenging decision-making problems. More concisely, it is a very good idea.

We thank the Reviewer for their thoughtful engagement with our manuscript. We appreciate their recognition of elasticity as a key dimension of control that has the potential to advance our understanding of psychopathology and healthy decision-making.

Starting with theory, the authors do not provide a strong formal characterization of the proposed notion of elasticity. There are existing, highly general models of controllability (e.g., Huys & Dayan, 2009; Ligneul, 2021) and the elasticity idea could naturally be embedded within one of these frameworks. The authors gesture at this in the introduction; however, this formalization is not reflected in the implemented model, which is highly task-specific.

Our formal definition of elasticity, detailed in Supplementary Note 1, naturally extends the reward-based and information-theoretic definitions of controllability by Huys & Dayan (2009) and Ligneul (2021). We now further clarify how the model implements this formalized definition (lines 156-159).

“Conversely, in the ‘elastic controllability model’, the beta distributions represent a belief about the maximum achievable level of control (𝑎_Control_, 𝑏_Control_) coupled with two elasticity estimates that specify the degree to which successful boarding requires purchasing at least one (𝑎_elastic≥1_, 𝑏_elastic≥1_) or specifically two (𝑎_elastic2_, 𝑏_elastic2_) extra tickets. As such, these elasticity estimates quantify how resource investment affects control. The higher they are, the more controllability estimates can be made more precise by knowing how much resources the agent is willing and able to invest (Supplementary Note 1).”

Moreover, the authors present elasticity as if it is somehow "outside of" the more general notion of controllability. However, effort and investment are just specific dimensions of action; and resources like money, strength, and skill (the "highly trained birke") are just specific dimensions of state. Accordingly, the notion of elasticity is necessarily implicitly captured by the standard model. Personally, I am compelled by the idea that effort and resource (and therefore elasticity) are particularly important dimensions, ones that people are uniquely tuned to. However, by framing elasticity as a property that is different in kind from controllability (rather than just a dimension of controllability), the authors only make it more difficult to integrate this exciting idea into generalizable models.

We respectfully disagree that we present elasticity as outside of, or different in kind from, controllability. Throughout the manuscript, we explicitly describe elasticity as a dimension of controllability (e.g., lines 70-72, along many other examples). This is also expressed in our formal definition of elasticity (Supplementary Note 1).

The argument that vehicle/destination choice is not trivial because people occasionally didn't choose the instructed location is not compelling to me-if anything, the exclusion rate is unusually low for online studies. The finding that people learn more from non-random outcomes is helpful, but this could easily be cast as standard model-based learning very much like what one measures with the Daw two-step task (nothing specific to control here). Their final argument is the strongest, that to explain behavior the model must assume "a priori that increased effort could enhance control." However, more literally, the necessary assumption is that each attempt increases the probability of success-e.g. you're more likely to get a heads in two flips than one. I suppose you can call that "elasticity inference", but I would call it basic probabilistic reasoning.

We appreciate the Reviewer’s concerns but feel that some of the more subjective comments might not benefit from further discussion. We only note that controllability and its elasticity are features of environmental structure, so in principle any controllability-related inference is a form of model-based learning. The interesting question is whether people account in their world model for that particular feature of the environment.

The authors try to retreat, saying "our research question was whether people can distinguish between elastic and inelastic controllability." I struggle to reconcile this with the claim in the abstract "These findings establish the elasticity of control as a distinct cognitive construct guiding adaptive behavior". That claim is the interesting one, and the one I am evaluating the evidence in light of.

In real-world contexts, it is often trivial that sometimes further investment enhances control and sometimes it does not. For example, students know that if they prepare more extensively for their exams they will likely be able to achieve better grades, but they also know that there is uncertainty in this regard – their grades could improve significantly, modestly, or in some cases, they might not improve at all, depending on the type of exams their study program administers and the knowledge or skills being tested. Our research question was whether in such contexts people learn from experience the degree to which controllability is elastic to invested resources and adapt their resource investment accordingly. Our findings show that they do.

The authors argue for CCA by appeal to the need to "account for the substantial variance that is typically shared among different forms of psychopathology". I agree. A simple correlation would indeed be fairly weak evidence. Strong evidence would show a significant correlation after *controlling for* other factors (e.g. a regression predicting elasticity bias from all subscales simultaneously). CCA effectively does the opposite, asking whether-with the help of all the parameters and all the surveys-one can find any correlation between the two sets of variables. The results are certainly suggestive, but they provide very little statistical evidence that the elasticity parameter is meaningfully related to any particular dimension of psychopathology.

We agree with the Reviewer on the relationship between elasticity and any particular dimension of psychopathology. The CCA asks a different question, namely, whether there is a relationship between psychopathology traits and task parameters, and whether elasticity bias specifically contributes to this relationship.

I am very concerned to see that the authors removed the discussion of this limitation in response to my first review. I quote the original explanation here:

- In interpreting the present findings, it needs to be noted that we designed our task to be especially sensitive to overestimation of elasticity. We did so by giving participants free 3 tickets at their initial visits to each planet, which meant that upon success with 3 tickets, people who overestimate elasticity were more likely to continue purchasing extra tickets unnecessarily. Following the same logic, had we first had participants experience 1 ticket trips, this could have increased the sensitivity of our task to underestimation of elasticity in elastic environments. Such underestimation could potentially relate to a distinct psychopathological profile that more heavily loads on depressive symptoms. Thus, by altering the initial exposure, future studies could disambiguate the dissociable contributions of overestimating versus underestimating elasticity to different forms of psychopathology.

The logic of this paragraph makes perfect sense to me. If you assume low elasticity, you will infer that you could catch the train with just one ticket. However, when elasticity is in fact high, you would find that you don't catch the train, leading you to quickly infer high elasticity eliminating the bias. In contrast, if you assume high elasticity, you will continue purchasing three tickets and will never have the opportunity to learn that you could be purchasing only one-the bias remains.

The authors attempt to argue that this isn't happening using parameter recovery. However, they only report the *correlation* in the parameter, whereas the critical measure is the *bias*. Furthermore, in parameter recovery, the data-generating and data-fitting models are identical-this will yield the best possible recovery results. Although finding no bias in this setting would support the claims, it cannot outweigh the logical argument for the bias that they originally laid out. Finally, parameter recovery should be performed across the full range of plausible parameter values; using fitted parameters (a detail I could only determine by reading the code) yields biased results because the fitted parameters are themselves subject to the bias (if present). That is, if true low elasticity is inferred as high elasticity, then you will not have any examples of low elasticity in the fitted parameters and will not detect the inability to recover them.

The logic the Reviewer describes breaks down when one considers the dynamics of participants’ resource investment choices. A low elasticity bias in a participant’s prior belief would make them persist for longer in purchasing a single ticket despite failure, as compared to a person without such a bias. Indeed, the ability of the experimental design to demonstrate low elasticity biases is evidenced by the fact that the majority of participants were fitted with a low elasticity bias (μ = .16 ± .14, where .5 is unbiased).

Originally, the Reviewer was concerned that elasticity bias was being confounded with a general deficit in learning. The weak inter-parameter correlations in the parameter recovery test resolved this concern, especially given that, as we now noted, the simulated parameter space encompassed both low and high elasticity biases (range=[.02,.76]). Furthermore, regarding the Reviewer's concern about bias in the parameter recovery, we found no such significant bias with respect to the elasticity bias parameter (Δ(Simulated, Recovered) = -.03, p=.25), showing that our experiment could accurately identify low and high elasticity biases.

The statistical structure of the task is inconsistent with the framing. In the framing, participants can make either one or two second boarding attempts (jumps) by purchasing extra tickets. The additional attempt(s) will thus succeed with probability p for one ticket and 2p – p^^^2 for two tickets; the p^^^2 captures the fact that you only take the second attempt if you fail on the first. A consequence of this is buying more tickets has diminishing returns. In contrast, in the task, participants always jumped twice after purchasing two tickets, and the probability of success with two tickets was exactly double that with one ticket. Thus, if participants are applying an intuitive causal model to the task, they will appear to "underestimate" the elasticity of control. I don't think this seriously jeopardizes the key results, but any follow-up work should ensure that the task's structure is consistent with the intuitive causal model.

We thank the Reviewer for this comment, and agree the participants may have employed the intuitive understanding the Reviewer describes. This is consistent with our model comparison results, which showed that participants did not assume that control increases linearly with resource investment (lines 677-692). Consequently, this is also not assumed by our model, except perhaps by how the prior is implemented (a property that was supported by model comparison). In the text, we acknowledge that this aspect of the model and participants’ behavior deviates from the true task's structure, and it would be worthwhile to address this deviation in future studies.

That said, there is no reason that this will make participants appear to be generally underestimating elasticity. Following exposure to outcomes for one and three tickets, any nonlinear understanding of probabilities would only affect the controllability estimate for two tickets. This would have contrasting effects on the elasticity estimated to the second and third tickets, but on average, it would not change the overall elasticity estimated. On the other hand, such a participant is only exposed to outcomes for two and three tickets, they would come to judge the difference between the first and second tickets too highly, thereby overestimating elasticity.

The model is heuristically defined and does not reflect Bayesian updating. For example, it overestimates maximum control by not using losses with less than 3 tickets (intuitively, the inference here depends on what your beliefs about elasticity). Including forced three-ticket trials at the beginning of each round makes this less of an issue; but if you want to remove those trials, you might need to adjust the model. The need to introduce the modified model with kappa is likely another symptom of the heuristic nature of the model updating equations.

Note that we have tested a fully Bayesian model (lines 676-691), but found that this model fitted participants’ choices worse.

You're right; saying these analyses provides "no information" was unfair. I agree that this is a useful way to link model parameters with behavior, and they should remain in the paper. However, my key objection still holds: these analyses do not tell us anything about how *people's* prior assumptions influence behavior. Instead, they tell us about how *fitted model parameters* depend on observed behavior. You can easily avoid this misreading by adding a small parenthetical, e.g.

Thus, a prior assumption that control is likely available **(operationalized by \gamma_controllability)** was reflected in a futile investment of resources in uncontrollable environments.

We thank the Reviewer for the suggestion and have added this parenthetical (lines 219, 225).
